# Comments and corrections on 3D modeling studies of locomotor muscle moment arms in archosaurs

**DOI:** 10.7717/peerj.1272

**Published:** 2015-10-08

**Authors:** Karl Bates, Susannah C.R. Maidment, Emma R. Schachner, Paul M. Barrett

**Affiliations:** 1 Department of Musculoskeletal Biology, University of Liverpool, Liverpool, United Kingdom; 2 Department of Earth Science and Engineering, Imperial College, London, United Kingdom; 3 Department of Veterinary Clinical Sciences, Louisiana State University, Baton Rouge, LA, United States of America; 4 Department of Earth Sciences, The Natural History Museum, London, United Kingdom

**Keywords:** Moment arms, Computational modeling, Archosaurs, Bipedalism, Locomotion, Quadrupedalism

## Abstract

In a number of recent studies we used computer modeling to investigate the evolution of muscle leverage (moment arms) and function in extant and extinct archosaur lineages (crocodilians, dinosaurs including birds and pterosaurs). These studies sought to quantify the level of disparity and convergence in muscle moment arms during the evolution of bipedal and quadrupedal posture in various independent archosaur lineages, and in doing so further our understanding of changes in anatomy, locomotion and ecology during the group’s >250 million year evolutionary history. Subsequent work by others has led us to re-evaluate our models, which revealed a methodological error that impacted on the results obtained from the abduction–adduction and long-axis rotation moment arms in our published studies. In this paper we present corrected abduction–adduction and long axis rotation moment arms for all our models, and evaluate the impact of this new data on the conclusions of our previous studies. We find that, in general, our newly corrected data differed only slightly from that previously published, with very few qualitative changes in muscle moments (e.g., muscles originally identified as abductors remained abductors). As a result the majority of our previous conclusions regarding the functional evolution of key muscles in these archosaur groups are upheld.

## Introduction

In recent years mathematical–computational approaches have become an increasingly popular tool for studying the functional anatomy and locomotor biomechanics of extinct animals (e.g., reviews in Hutchinson & Allen, 2009; Hutchinson, 2011; Bates, 2013; Maidment & Barrett, 2014). Modeling methods are particularly important because they allow the function of unique morphological structures seen in extinct animals to be investigated directly, without need for reference to analogous living taxa (Bates et al., 2010). This is particularly appealing in the case of animals such as non-avian dinosaurs that lack direct morpho-functional analogues among living animals (Hutchinson & Allen, 2009; Hutchinson, 2011). For this reason, such computational approaches have been applied extensively to non-avian dinosaurs, which possessed body shapes, sizes and skeletal morphologies dissimilar to those of extant terrestrial vertebrates (Hutchinson & Allen, 2009; Maidment et al., 2014). Dinosaurs radiated into a diverse array of body shapes and sizes during their long (>150 million year) evolutionary history, and, for example, underwent several evolutionary transitions between obligate bipedalism and quadrupedalism (e.g., Maidment & Barrett, 2012; Maidment & Barrett, 2014). Living birds are the direct descendants of theropod dinosaurs and thus the fossil record of this group provides direct evidence of the morphological and potential functional changes that occurred during the evolution of their terrestrial, aerial and aquatic styles of locomotion (e.g., Hutchinson & Allen, 2009; Allen et al., 2013).

In several recent studies (Bates & Schachner, 2012; Bates et al., 2012; Maidment et al., 2014; Maidment, Bates & Barrett, 2014) we used computer modeling to investigate the evolution of muscle leverage (moment arms) and function in various archosaur lineages. Specifically, we sought to quantify the level of disparity and convergence in muscle moment arms among bipedal archosaurs, namely poposaurids (bipedal crurotarsans) and various ornithischians and non-avian theropods, and to draw inferences on how muscle leverage and recruitment during gait may have changed during the evolution of the flexed femoral postures and long-axis rotation-based mode of lateral limb support characteristic of extant birds (Bates & Schachner, 2012; Bates et al., 2012). In further work we employed the same modeling approach to examine changes in muscle moment arms during ornithischian evolution, with a particular focus on the evolution of quadrupedality within the group (Maidment et al., 2014; Maidment, Bates & Barrett, 2014). Ornithischian dinosaurs were primitively bipedal with forelimbs modified for grasping, but quadrupedalism evolved in the clade on at least three independent occasions (Maidment & Barrett, 2012). By building and analyzing models of eight exemplar ornithischian taxa we were able to quantify similarities and differences among individual taxa, between quadrupedal and bipedal taxa, and among taxa representing the three major ornithischian lineages (Thyreophora, Ornithopoda, Marginocephalia).

**Figure 1:**
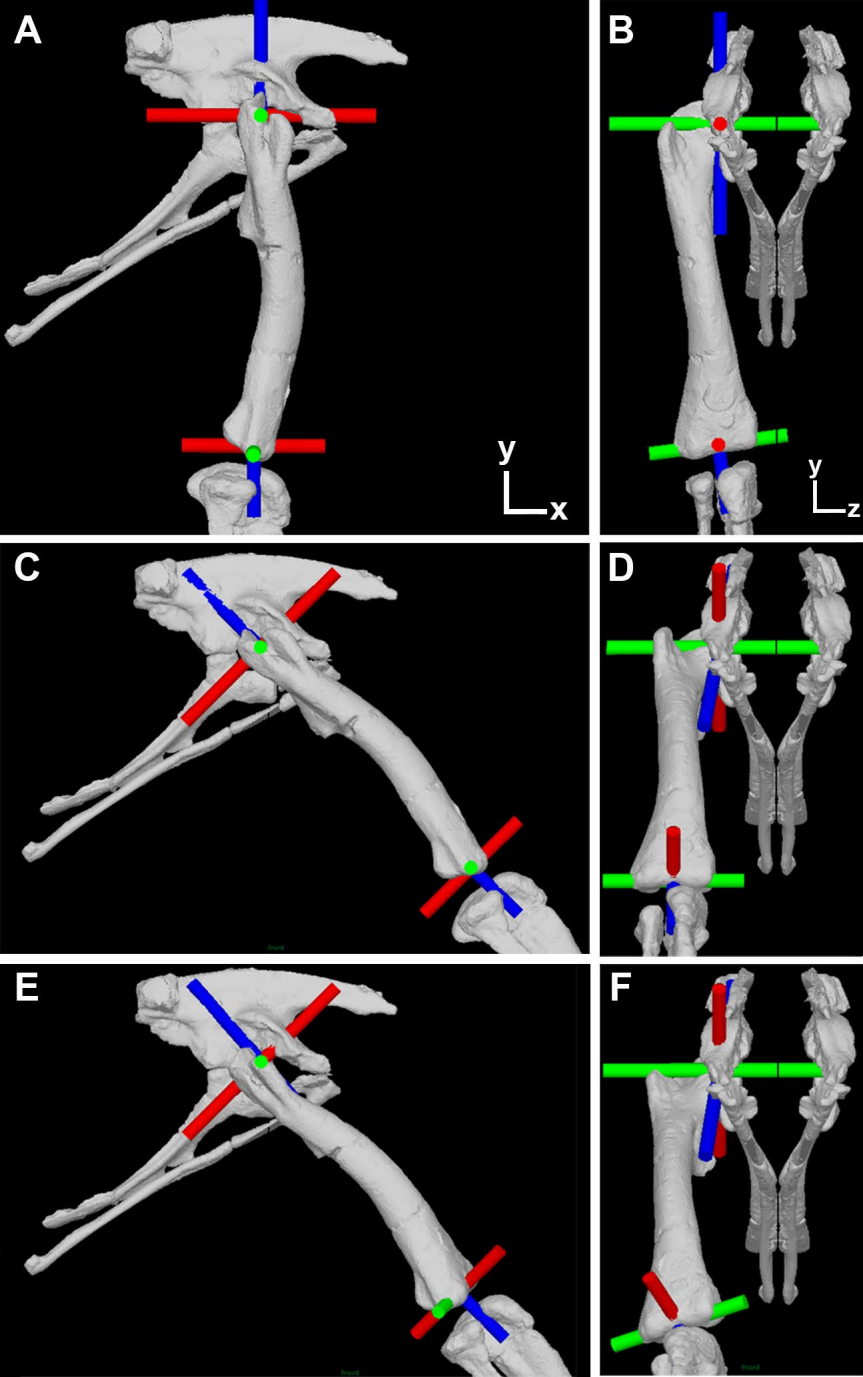
Illustration of the corrected joint axes in the *Lesothosaurus* model. Model shown in (A) lateral and (B) cranial views in the initial ‘neutral’ posture with all hip joint axis at zero. Model shown in (C) lateral and (D) cranial views with hip flexion-extension at 45° flexion and the femur abducted by 10°, but long-axis rotation axis at zero. Model shown in (E) lateral and (F) cranial views with hip flexion-extension at 45° flexion and the femur abducted by 10° and laterally rotated about the long-axis rotation axis by 25°. Flexion-Extension axes are shown in green, abduction–adduction in red and long-axis rotation in blue. The global co-ordinate system axes are indicated in (A) and (B).

Recently, Hutchinson et al. (2014) compared moment arm predictions from our ostrich musculoskeletal model (Bates & Schachner, 2012) to their own model, which was developed using a more exhaustive experimental protocol. This study found a strong match between the flexion-extension moment arms of the two models, but noted systematic differences between the abduction–adduction and long-axis rotation moment arms of some hip muscles (Hutchinson et al., 2014). Re-inspection of our models has revealed that these differences are due to a coding error, one that is present in all of our published models (Bates & Schachner, 2012; Bates et al., 2012; Maidment et al., 2014; Maidment, Bates & Barrett, 2014). The error relates to the specification of joint axes in the models: as the posture of the models were modified to output moment arms across a range of hip joint angles, the axes of the hip joint remained in its initial orientation rather than rotating with the femoral segment and knee joint of the models (Fig. 1), due to joint axes being specified relative to the static trunk segment rather than the mobile thigh segment. Thus the abduction–adduction and long-axis rotation data presented in our previous study represents muscle moment arms calculated about anatomically and functionally inappropriate axes. Hutchinson et al. (2015) subsequently compared their data to our corrected abduction–adduction and long-axis rotation moment arms and found a much stronger agreement between the models.

The purpose of this paper is to present corrected abduction–adduction and long-axis rotation moment arms for all our models (Fig. 1), and to evaluate the impact of this new data on the conclusions of our previous studies (Bates & Schachner, 2012; Bates et al., 2012; Maidment et al., 2014; Maidment, Bates & Barrett, 2014).

## Materials and Methods

Our protocol follows our previous studies exactly, apart from the necessary correction made to the hip joint axes. We recommend Bates et al. (2012) and Maidment et al. (2014) for detailed descriptions of our prior methodology. As stated above, the error in our previous studies relates to the specification of all joint hip axes in the models relative to pelvic segment. This is appropriate only for the hip flexion-extension axis, which should (and did) remain parallel to the *z*-axis (left–right or medial-lateral) axis of the pelvis during all three-dimensional rotations of the femur (Fig. 1). In our corrected models the hip abduction–adduction axis initially lies parallel to the global (and pelvic) *x*-axis and the long-axis rotation axis lies parallel to the global (and pelvic) *y*-axis in the neutral posture (i.e., all angles at zero; Figs. 1A and 1B). As the hip is flexed (or extended) the hip abduction–adduction and long-axis axes rotate with the femur (Fig. 1C), while femoral rotation about the hip abduction–adduction axis causes rotation of the long-axis rotation joint axis but not the flexion-extension joint axis (Fig. 1D). Long-axis rotation of the femur does not rotate the flexion-extension or abduction–adduction axes (Figs. 1E and 1F).

To extract corrected abduction–adduction and long-axis rotation muscle moment arms across a spectrum of postures we manipulated the orientation of the thigh segment relative to the pelvis and the orientation of a single hinge joint at the hip. As in our previous studies, the thigh segment was started at an orientation that placed the hip at 10° of abduction in all models. We then produced several iterations of our model in which hip flexion-extension varied in 15-degree increments to sample a spectrum of hip flexion-extension angles. At each flexion-extension posture two simulations were carried out; firstly with the axis of the hinge joint orientated such that only pure abduction–adduction joint rotation could occur at the hip; and secondly with the axis of the hinge joint orientated such that only pure long-axis rotation could occur at the hip. Orientation of the hinges in these two model iterations followed the joint axes rotation scheme noted above (Fig. 1) and for our model manipulations it was simply necessary to rotate the axes by the same magnitude applied to alter femoral flexion-extension posture. Example animations are provided to illustrate this process (Movies S1–S5). To extract muscle moment arms the appropriate muscles were activated to induce rotation of the hinge joint. Muscle moment arms were then calculated from joint angle change and muscle length change as in our previous studies.

## Results and Discussion

All corrected moment arm data for hip abduction–adduction and long-axis rotation are tabulated in the supplementary material. In general our new corrected data differ only slightly from our original data, and there were very few qualitative changes in muscle moments (e.g., muscles originally identified as abductors remained abductors). Rather than describe every quantitative change in moment arm values we focus on the muscles relevant to the hypotheses and conclusions in each of our previous studies. For clarity we address each study individually in the following sections.

### Bates & Schachner (2012): moment arms and muscle function *Poposaurus* and in bipedal ornithodirans

**Figure 2:**
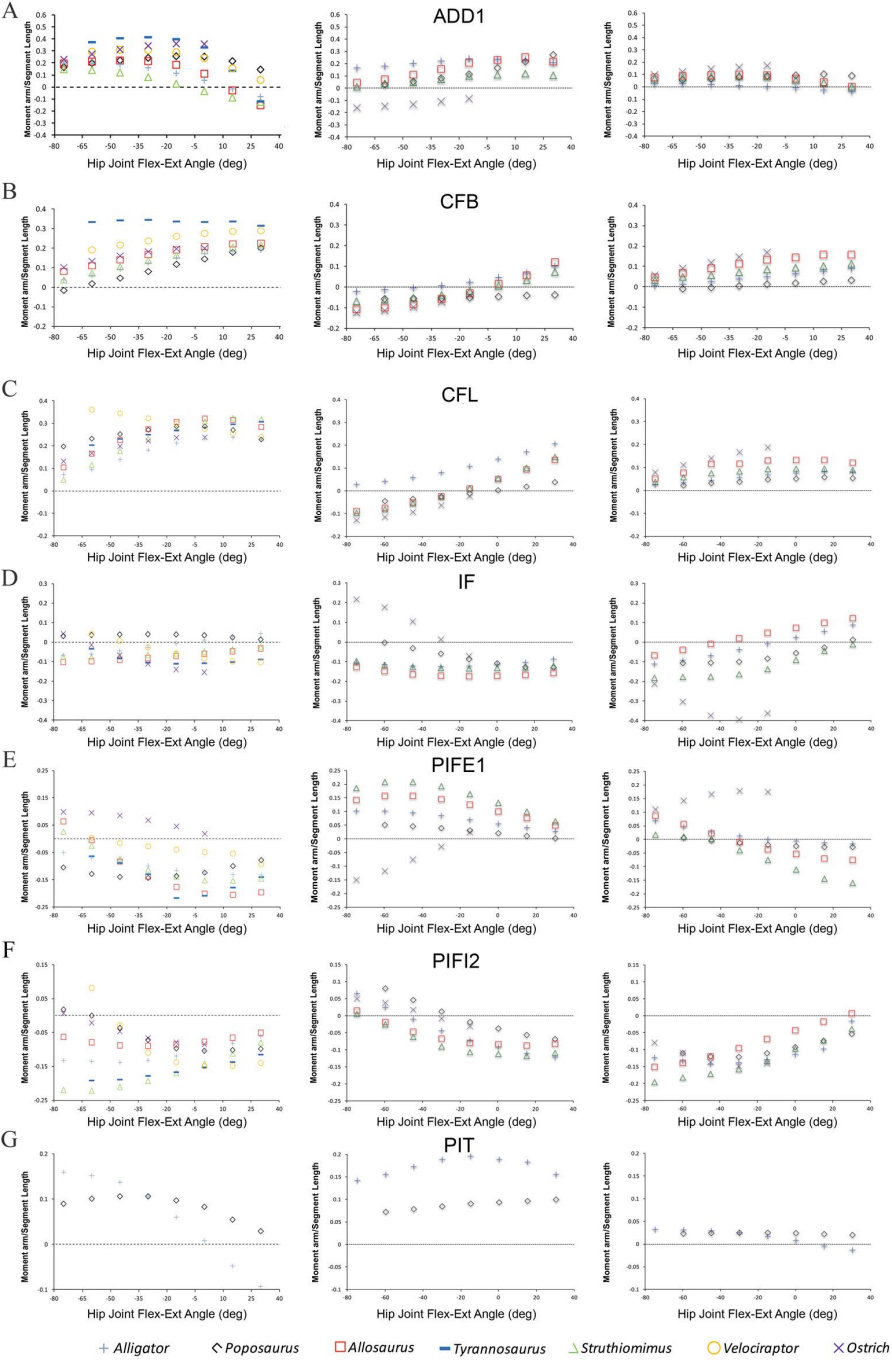
Predicted pelvic muscle moment arms for hip flexion-extension (left), and corrected data for abduction–adduction (centre) and long-axis rotation (right) in key muscle groups (A) ADD1, (B) CFB, (C) CFL, (D) IF, (E) PIFE1, (F) PIFI2 and (G) PIT over a range of hip joint flexion-extension angles. All data normalized by femoral length. Only flexion-extension data are available for *Tyrannosaurus* and *Velociraptor* from previous studies (Hutchinson et al., 2005; Hutchinson et al., 2008), while PIT is present only in *Poposaurus* and *Alligator*, having been lost in ornithodirans.

The majority of the functional and evolutionary conclusions drawn by Bates & Schachner (2012) are upheld because the corrected moment arm magnitudes differ only slightly from our published values, and because the correction tends to affect all taxa equally and, therefore, the relative differences and similarities between the models have been preserved (Fig. 2).

**Figure 3:**
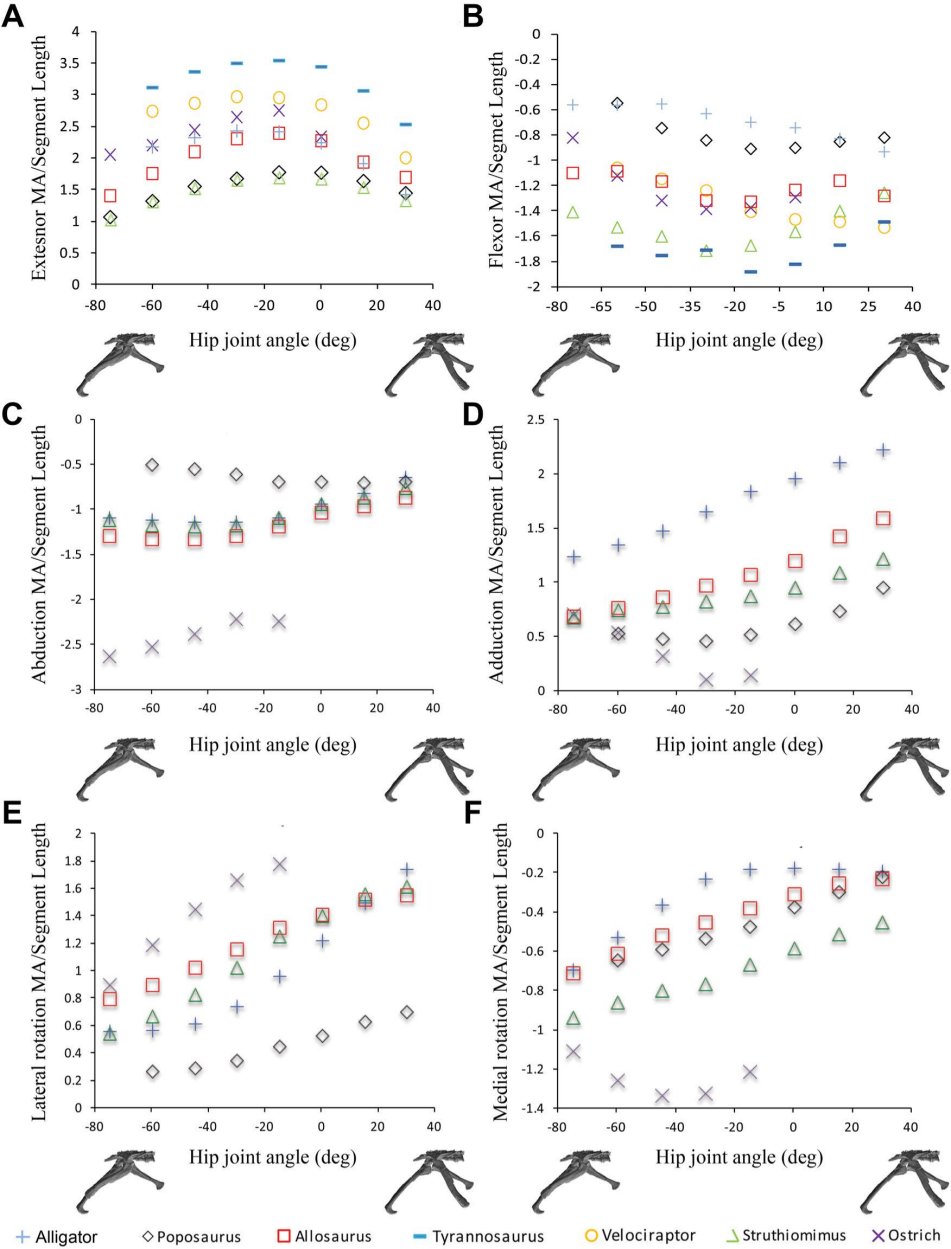
Sum of (A) hip extensor, (B) hip flexor, (C) hip abduction, (D) adduction, (E) lateral femoral rotation and (F) medial femoral rotation muscle moment arms normalized by segment length for *Poposaurus, Alligator* and ornithodiran bipeds. All data normalized by femoral length. Only flexion-extension data are available for Tyrannosaurus and Velociraptor from previous studies (Hutchinson et al., 2005; Hutchinson et al., 2008).

Bates & Schachner (2012) concluded that muscle moment arm polarities and joint angle relationships in key hip muscle groups (Hutchinson & Gatesy, 2000) are generally conservative despite the shifts in skeletal architecture, posture, body size and locomotor behaviour covered by our sampled taxa. This suggests that muscle origins and insertions remained relatively stable with respect to the hip joint across Archosauria. This conclusion is supported by our corrected data (Fig. 2). Functional interpretations regarding specific key muscles, notably the iliofemoralis (IF) and puboischiofemoralis externus (PIFE), also retain their support. Specifically, the cranial portion of the IF group has a much larger medial rotation moment arm in the ostrich (Fig. 2D), while PIFE1 and 2 extend the hip and rotate the femur laterally in the ostrich, but are hip flexors and medial rotators in all other taxa (Fig. 2E). Our summary observations that abduction–adduction and long-axis rotation moment arms are consistently low in *Poposaurus*, whereas adduction moment arms are generally low in the ostrich but high in *Alligator* (Figs. 2 and 3) are still supported. However, two changes are notable, although neither impacts the main conclusions of our previous study. Previously, the caudofemoralis brevis (CFB) muscle was considered a femoral abductor in *Poposaurus*, but an adductor in all other taxa (Fig. 3B in Bates & Schachner, 2012). Our corrected data shows that while CFB remains an obligatory abductor in *Poposaurus*, its function changes with joint angle in all other models, such that this muscle exerts an abductor moment at flexed postures and an adductor moment at extended postures (Fig. 2B). The other notable change is the overall reduction in medial long-axis rotation moment arms in the *Alligator* model, which have dropped below the magnitudes predicted for *Poposaurus* (Fig. 3).

### Bates et al. (2012): moment arms and muscle function in *Lesothosaurus* and bipedal ornithodirans

In this study we first presented detailed analyses of muscle moment arms in the basal, bipedal ornithischian dinosaur *Lesothosaurus*, and subsequently compared these data to a range of other dinosaurs (models from Bates & Schachner (2012)). We follow the same protocol here, first highlighting changes to our *Lesothosaurus* model, and then discussing changes to our comparative analysis.

**Figure 4:**
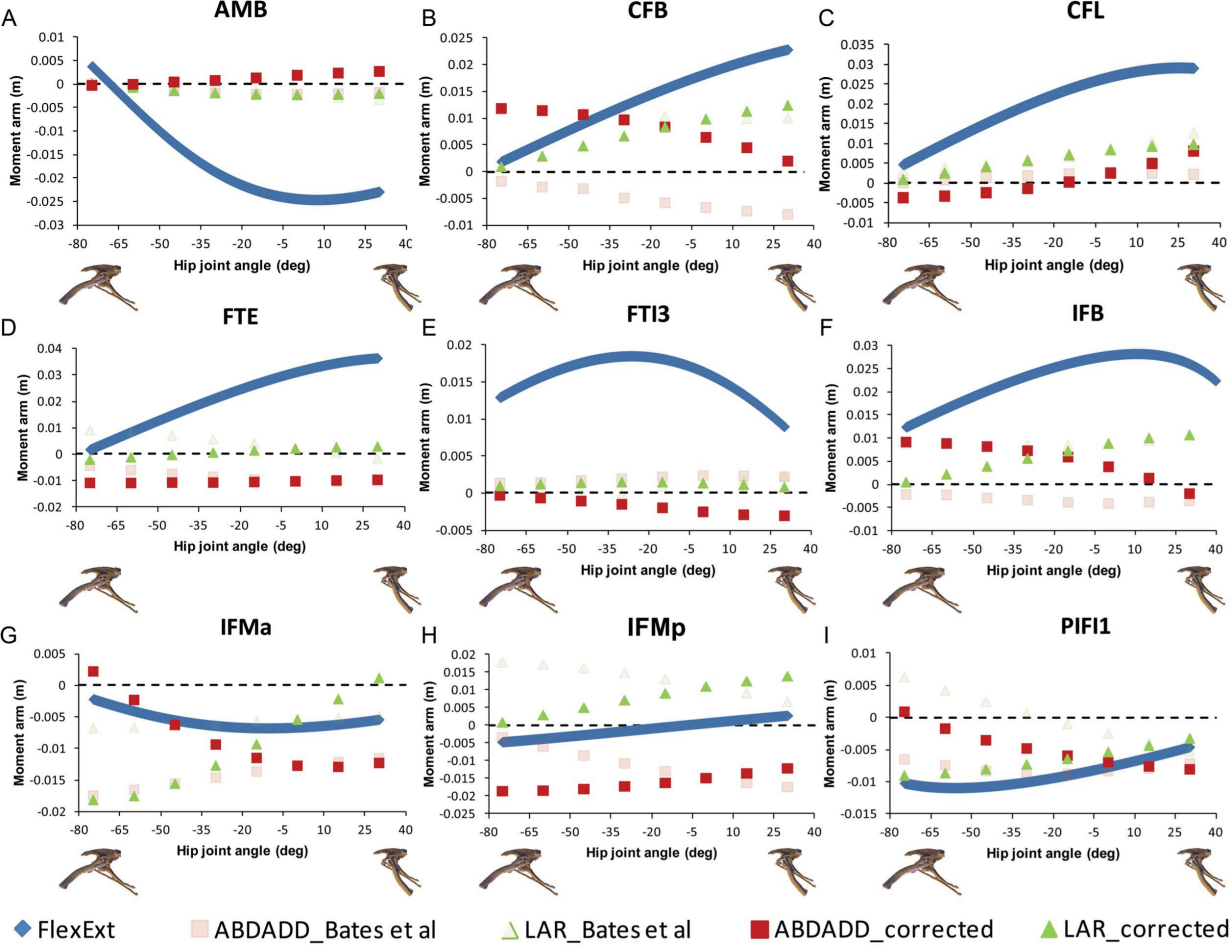
Corrected hip muscle moment arm predictions for (A) AMB, (B) CFB, (C) CFL, (D) FTE, (E) FTI3, (F) IFB, (G) IFMa, (H) IFMp and (I) PIFI1 for a range of hip flexion-extension angles in *Lesothosaurus*. A positive hip joint angle (*x*-axis) indicates hip extension (femoral retraction), while a negative hip joint angle indicates hip flexion (femoral protraction), as shown by the small images of the pelvis of *Lesothosaurus* in the left lateral view along the *x*-axis of each graph. A negative moment arm (*y*-axis) for flexion-extension is a moment arm for flexion; a negative moment arm for abduction–adduction is a moment arm for abduction; a negative moment arm for long axis rotation is a moment arm for medial rotation. FlexExt, flexion-extension; ABDADD, abduction–adduction; LAR, long axis rotation.

**Figure 5:**
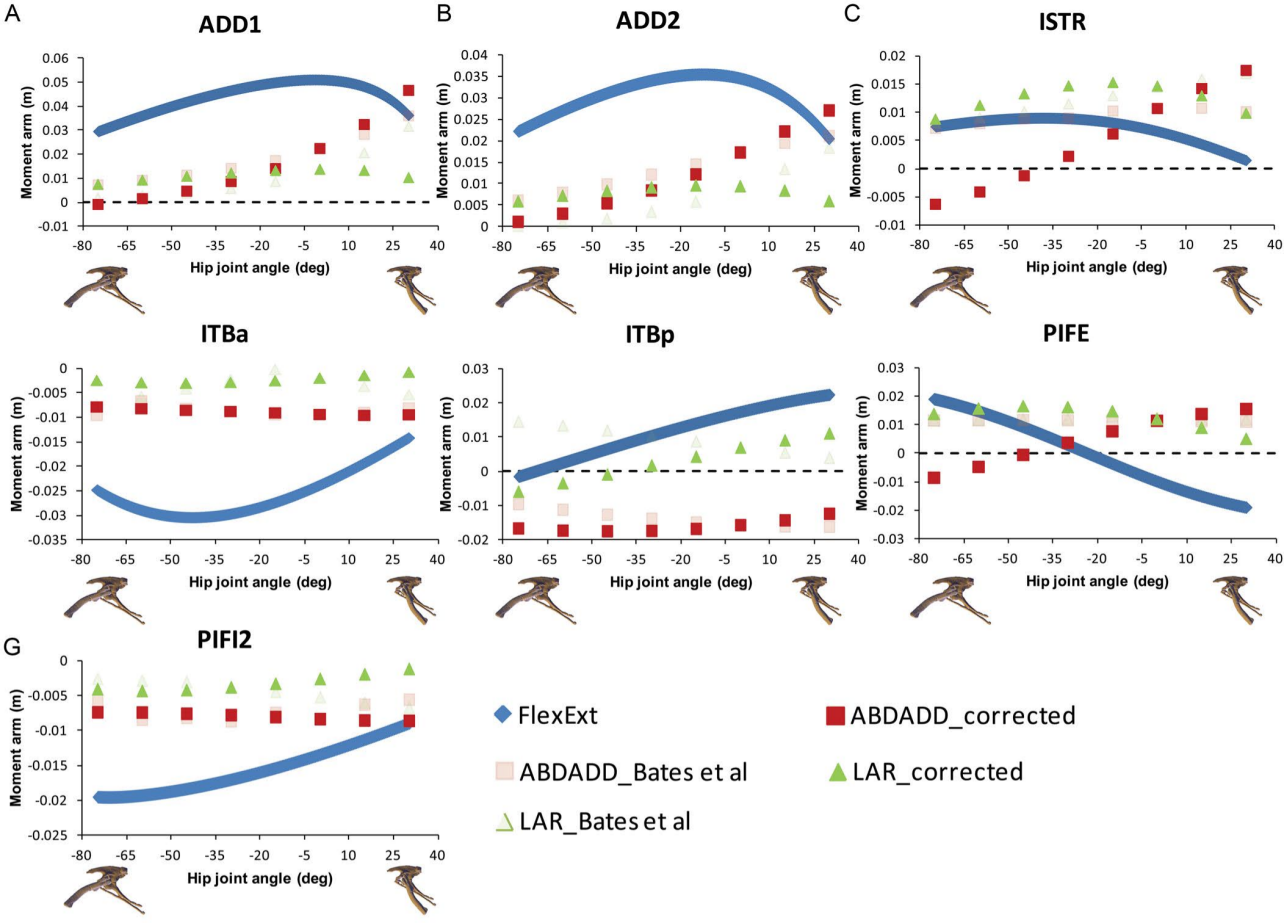
Corrected hip muscle moment arm predictions for (A) ADD1, (B) ADD2, (C) ISTR, (D) ITBa, (E) ITBp, (F) PIFE, (G) PIFI2, the muscles for which the sensitivity analysis was performed, for a range of hip flexion-extension angles in *Lesothosaurus*. A positive hip joint angle (*x*-axis) indicates hip extension (femoral retraction), while a negative hip joint angle indicates hip flexion (femoral protraction), as shown by the small images of the pelvis of *Lesothosaurus* in the left lateral view along the *x*-axis of each graph. A negative moment arm (*y*-axis) for flexion-extension is a moment arm for flexion; a negative moment arm for abduction–adduction is a moment arm for abduction; a negative moment arm for long axis rotation is a moment arm for medial rotation. FlexExt, flexion-extension; ABDADD, abduction–adduction; LAR, long axis rotation.

Corrections to our original analyses indicate that several muscles in *Lesothosaurus* incur minor changes in abduction–adduction moment arms. The caudofemoralis longus (CFL) and ischotrochantericus muscles (ISTR) and PIFE group were previously found to maintain adduction moment arms across the range of postures tested (Figs. 4 and 5 in Bates et al. (2012)). Our corrected data suggest that these muscles exerted an adduction moment at extended postures, but switched to an abduction moment at flexed postures (Figs. 4 and 5; but see Bates et al. (2012) for discussion of uncertainty in predictions for PIFE and ISTR muscles). The angular dependency of cranial portion of the IF group is also reversed in our corrected data: the muscle is now predicted to be a weak adductor at flexed postures and a weak abductor at extended postures (Fig. 4).

Modest changes to femoral long-axis rotation moment arms in the *Lesothosaurus* model were also found. The long-axis rotation moment arms of the adductor femoris (ADD1 and ADD2) muscles now show only very modest postural dependency (Fig. 5). The same applies to the PIFI1 muscle (Fig. 4) and cranial portion of the iliotibialis group (ITBa, or IT1 in other studies), which are now found to be medial rotators at all hip joint angles tested (Fig. 5). Three muscles incurred reversals in the angular or postural dependency of their long-axis rotation moment arms. The femorotibialis externus (FTE) and caudal portion of iliotibialis group (ITBp) muscles are now found to be weak medial rotators at flexed postures and weak lateral rotators at extended postures (Figs. 4 and 5). The caudal portion of the IF group is now found to be a weak lateral rotator at flexed postures, with an increasingly larger lateral rotation moment arm as the hip is extended (Fig. 4).

**Figure 6:**
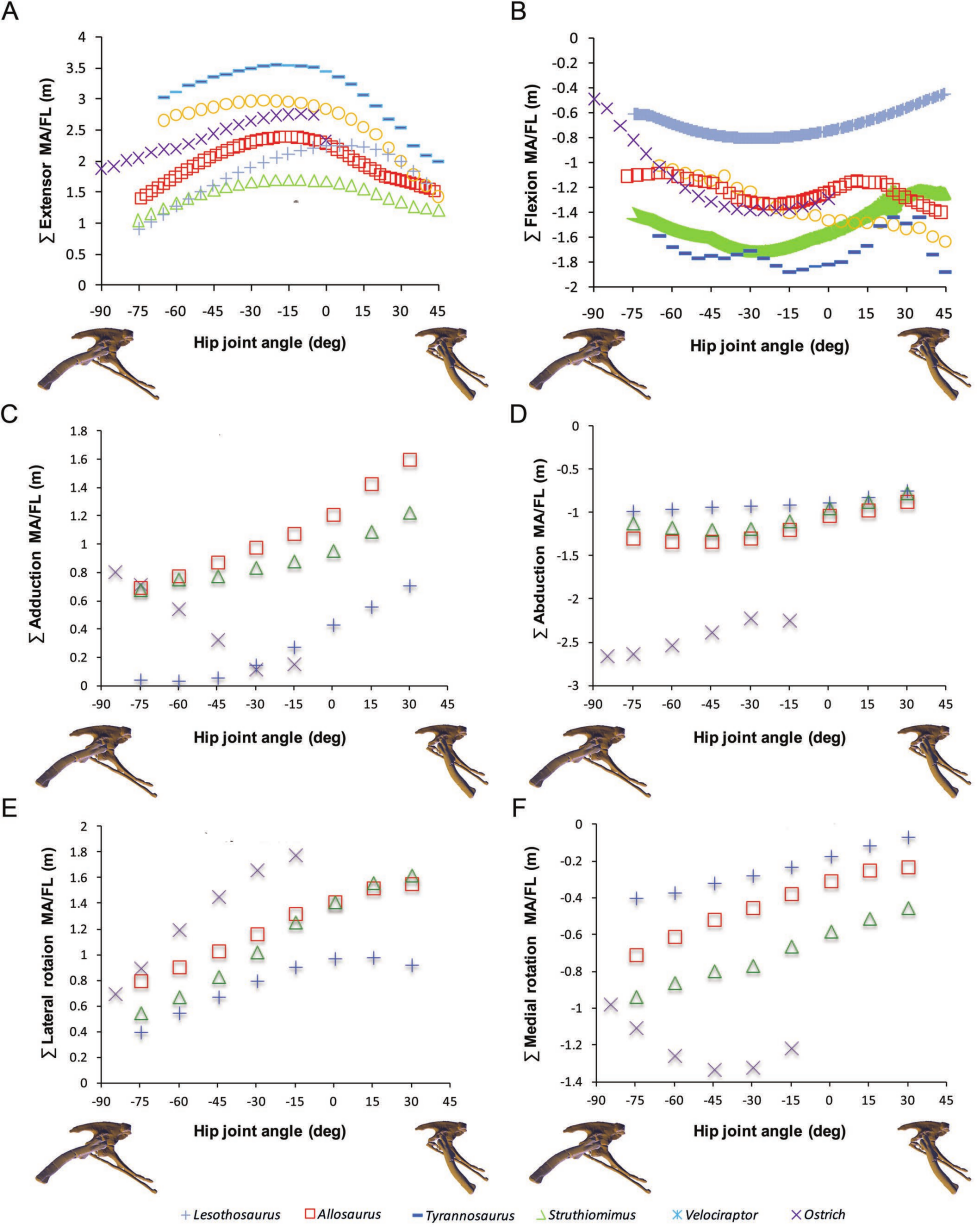
Corrected data for sum of (A) hip extensor, (B) hip flexor, (C) adduction, (D) abduction, (E) lateral femoral rotation and (F) medial femoral rotation muscle moment arms normalized by segment length for *Lesothosaurus* and other dinosaurian bipeds.

In our comparative analysis we made a number of statements regarding comparative trends in our moment arm data. We stated that summed adductor moment arms decreased slightly with hip flexion (Fig. 6C in Bates et al. (2012)) in all non-avian dinosaurs, while summed abductor moment arms increased slightly with hip extension (based on Fig. 6D in Bates et al. (2012)). Adduction moment arms remained relatively unaffected by our corrected joint axes, although abduction moment arms now tend to decrease slightly with increasing hip extension (Fig. 6). Our conclusion that *Lesothosaurus* and the ostrich had low summed moment arms for adduction, while those of *Struthiomimus* and *Allosaurus* were higher (Fig. 6C in Bates et al. (2012)) remains valid, as does our inference that *Lesothosaurus* had the lowest summed abductor moment arms, with non-avian theropods exhibiting intermediate summed abductor moment arms, and the ostrich showing the highest values (Figs. 6C and 6D in Bates et al. (2012); Fig. 6). We previously stated that summed moment arms for both adduction and abduction decreased slightly with hip flexion in the ostrich model (based on Figs. 6C and 6D in Bates et al. (2012)). This statement represents a typographic error, as our previous analyses (and with our corrected data; Fig. 6) showed that adduction moment arms increased with increasing hip flexion. In our corrected data abduction moment arms also increase with increasing hip flexion (Fig. 6).

We previously concluded that summed long-axis rotator moment arms displayed a taxonomic signal with hip flexion and extension (Figs. 6E and 6F in Bates et al. (2012)). *Lesothosaurus* had an extremely weak summed medial rotator moment arm (Fig. 6F in Bates et al. (2012)) compared with those of theropods, while its summed moment arm for lateral rotation (Fig. 6E in Bates et al. (2012)) was similar to that of non-avian theropods in magnitude. The ostrich had significantly higher summed medial and lateral rotator moment arms than any other dinosaur modeled (Figs. 6E and 6F in Bates et al. (2012)). These conclusions are still supported by our corrected data, although *Lesothosaurus* now shows weaker summed lateral rotation moment arms compared to other taxa (Fig. 6).

There are few changes of note in our comparative analysis of individual muscles. As in Bates & Schachner (2012), our inferences regarding CFB require adjustment. We previously concluded that CFB has an adduction moment arm in the theropods examined, while it has an abduction moment in *Lesothosaurus* (Fig. 7B in Bates et al. (2012)), but this distinction is no longer supported in our corrected data. We also previously stated that PIFE has a lateral rotator moment in the ostrich and *Lesothosaurus*, while it has a medial rotator moment arm in the non-avian theropods (Figs. 9C and 9F in Bates et al. (2012)). This statement largely holds following analysis of the corrected data (Figs. 2 and 4), although the non-avian theropod models do have weak lateral rotation moment arms at postures >45° hip flexion. However, the conclusion that *Lesothosaurus* and the ostrich have similar moment arms for this muscle, and that both of these are different from those of non-avian theropods, is still supported (Figs. 2 and 4).

### Maidment, Bates & Barrett (2014) and Maidment et al. (2014): moment arms and muscle function evolution in ornithischian dinosaurs

Maidment, Bates & Barrett (2014) presented a preliminary analysis of muscle moment arm evolution in ornithischian dinosaurs, which was subsequently expanded upon in Maidment et al. (2014) by the addition of five further models. We therefore discuss the implications of our corrected data for both studies synchronously.

**Figure 7:**
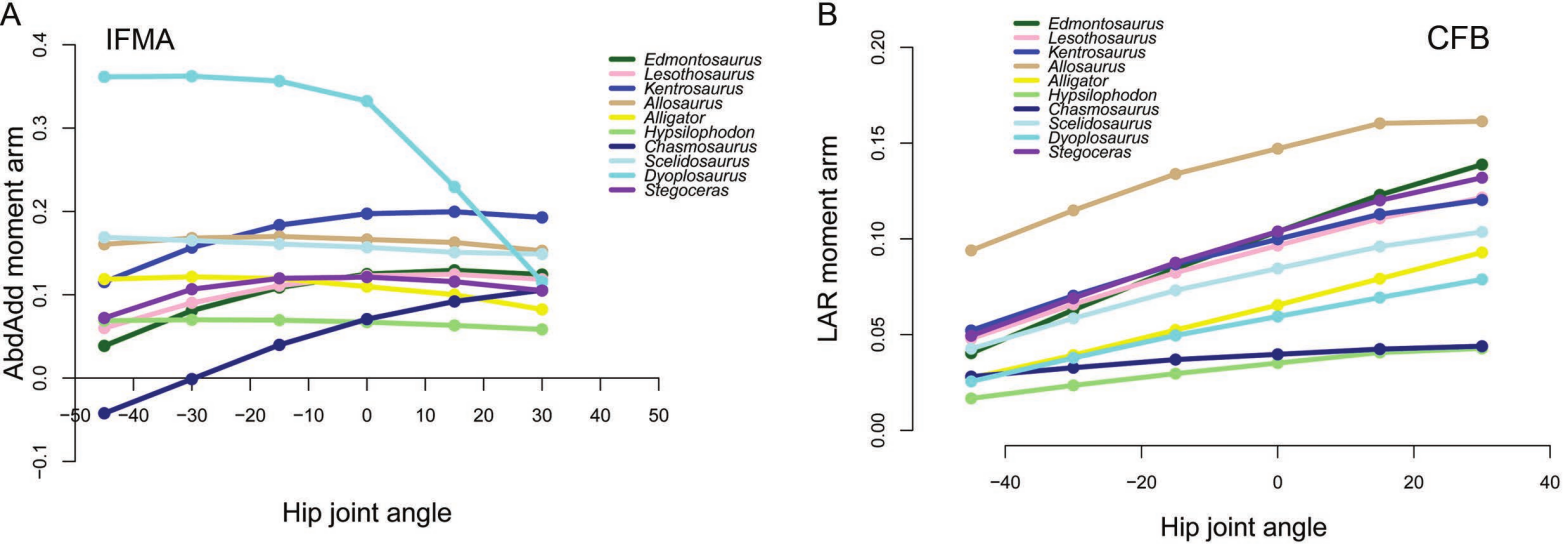
Examples of generally lower moment arms in *Chasmosaurus* and *Hypsilophodon* in our corrected data. Negative values are adduction, medial rotation and flexion, while positive values correspond with abduction, lateral rotation and extension. (A) Abduction-adduction moment arms for IFMA across a range of hip flexion-extension angles; (B) Long axis rotation moment arms for CFB across a range of hip flexion-extension angles.

**Figure 8:**
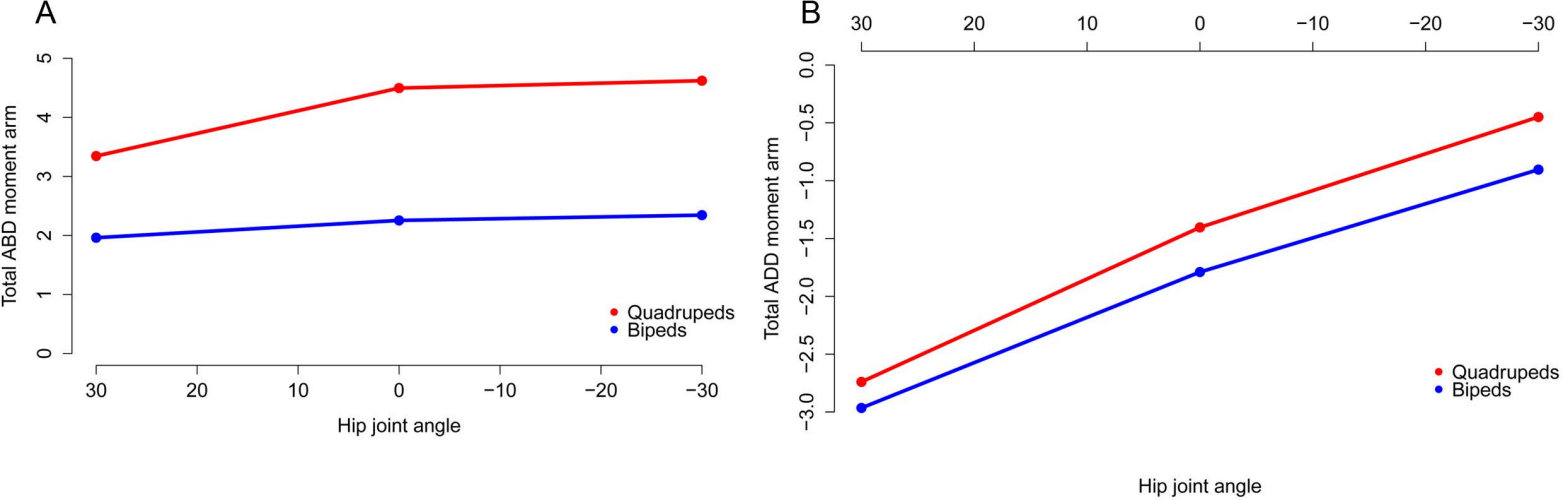
Summed abduction (A) and adduction (B) moment arms for quadrupeds (*Kentrosaurus, Dyoplosaurus, Chasmosaurus*) and bipeds (*Lesothosaurus, Hypsilophodon, Stegoceras*) across a range of hip flexion-extension angles. All moment arms are normalized by femoral length. Negative values are adduction and flexion, while positive values correspond with abduction and extension.

**Figure 9:**
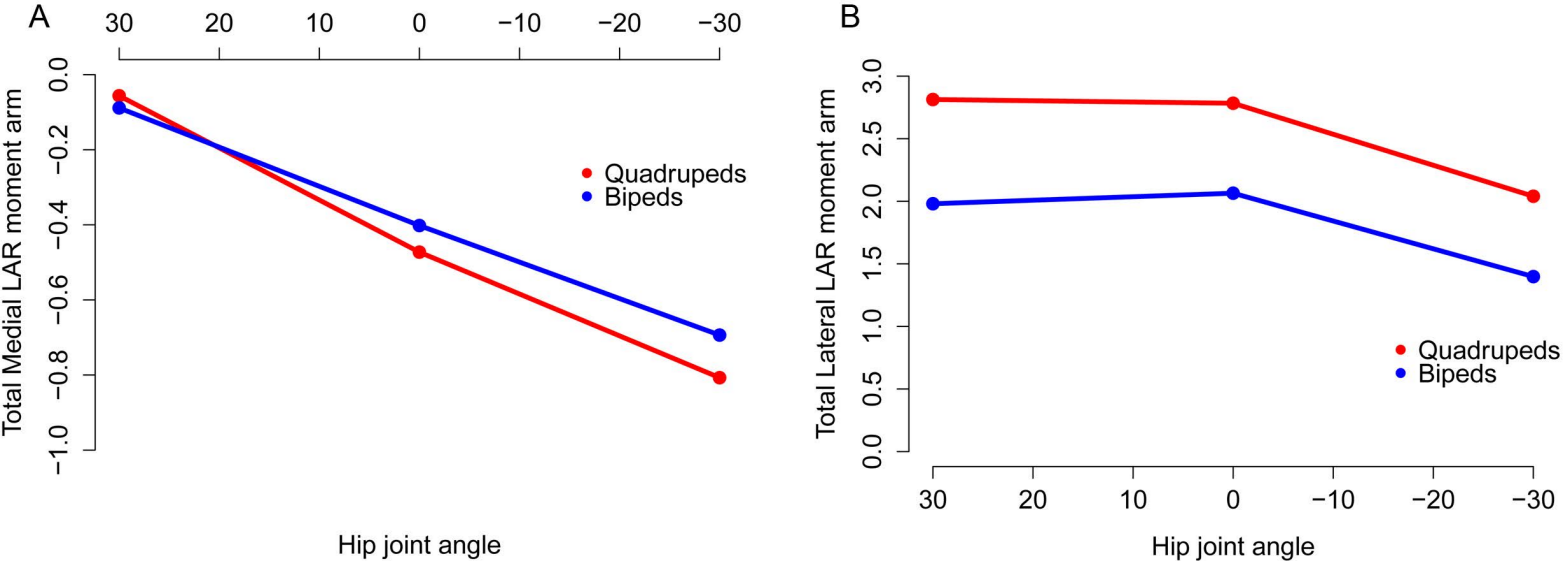
Summed medial (A) and lateral (B) long axis rotation moment arms for quadrupeds (*Kentrosaurus, Dyoplosaurus, Chasmosaurus*) and bipeds (*Lesothosaurus, Hypsilophodon, Stegoceras*) across a range of hip flexion-extension angles. All moment arms are normalized by femoral length. Negative values are medial rotation and flexion, while positive values correspond with lateral rotation and extension.

**Figure 10:**
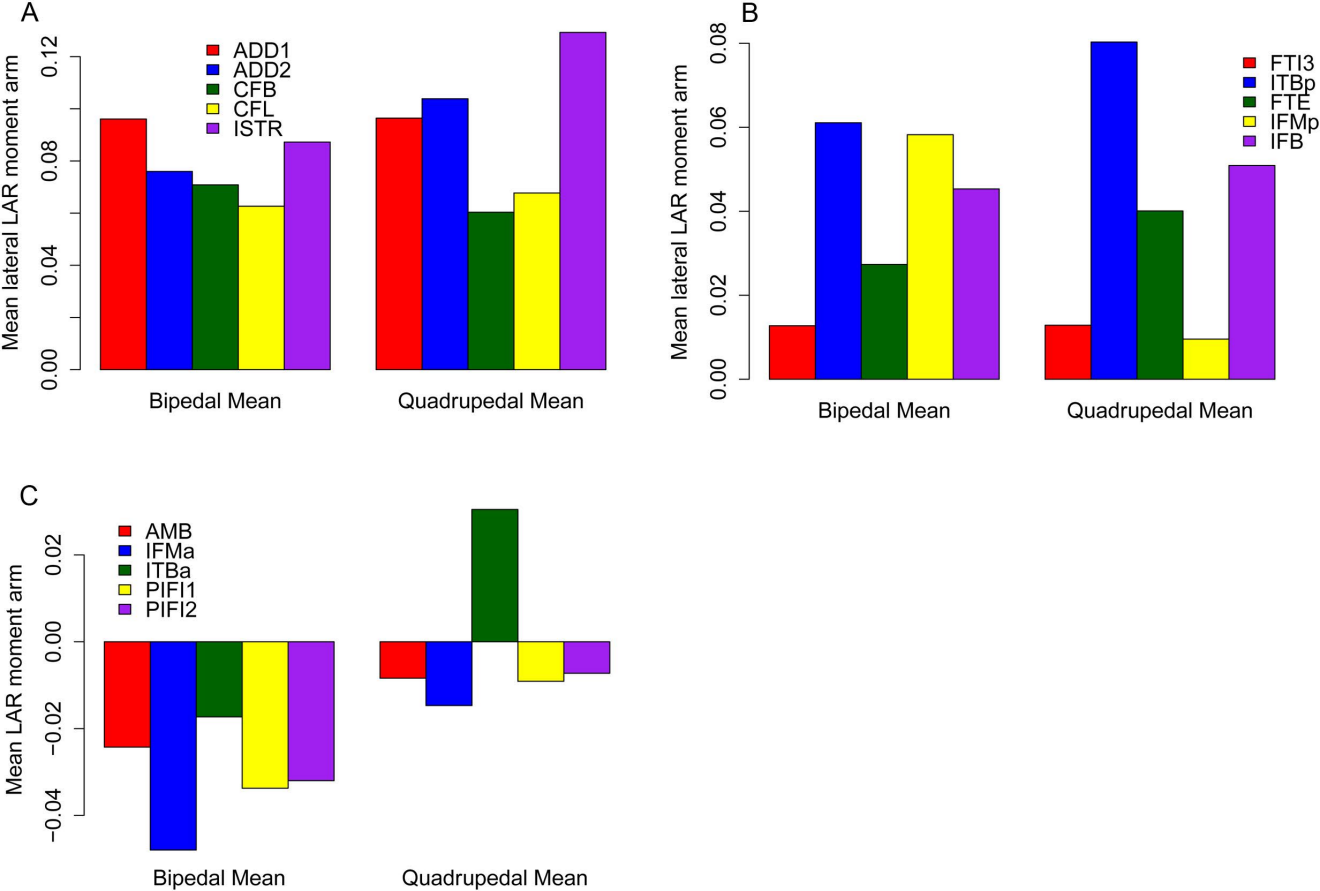
Mean long axis rotation moment arms for quadrupeds (*Kentrosaurus, Dyoplosaurus, Chasmosaurus*) and bipeds (*Lesothosaurus, Hypsilophodon, Stegoceras*). All moment arms are normalized by femoral length. Negative values are medial rotation, while positive values correspond with lateral rotation.

**Figure 11:**
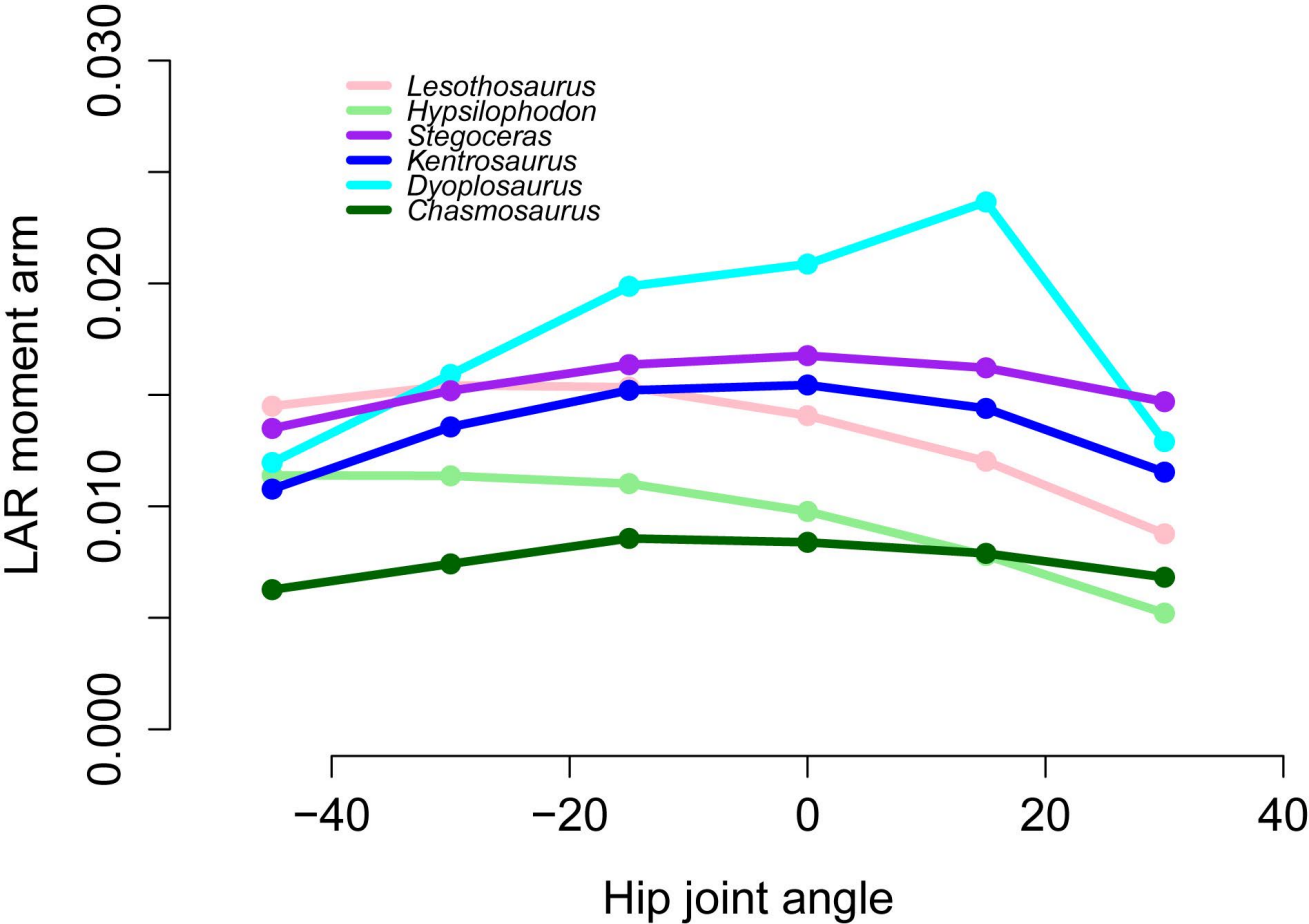
Lateral rotator moment arms across a range of hip flexion-extension angles for FTI3.

The majority of our original conclusions regarding muscle moment arm and functional evolution within Ornithischia are still supported by analysis of our corrected data. *Chasmosaurus* and *Hypsilophodon* are still distinguished by low moment arms for many muscles and functions (e.g., Fig. 7). Quadrupedal taxa (*Kentrosaurus, Dyoplosaurus, Chasmosaurus*) are also found to maintain higher abductor (Fig. 8A) but lower adductor (Fig. 8B) moment arms than bipedal taxa (*Lesothosaurus, Hypsilophodon, Stegoceras*), as found previously. In our original data, differences between long-axis rotation moment arms in quadrupeds and bipeds were not clear. There was little difference when summed medial rotation moment arms were examined, and this observation remains the case (Fig. 9A). A much greater difference between quadrupeds and bipeds is observed, however, when lateral rotation moment arms are examined (Fig. 9B). In our original data, there was some suggestion that lateral rotator moment arms were greater among quadrupedals, and this is more strongly supported by our corrected data. Our corrected data also supports our original assertion that bipeds appear to have higher mean medial rotation moment arms than quadrupeds (Fig. 10). The one other change of note occurs in the long-axis rotation moment arm of the flexor tibialis internus 3 (FTI3) muscle. We previously concluded that FTI3 was a lateral rotator at high flexion angles but became a medial rotator at high extension angles in quadrupeds and the reverse in bipeds (Maidment et al., 2014). In our corrected data (Fig. 11) there is no difference in the action of FTI3 between quadrupeds and bipeds, and it is a lateral rotator at all hip angles in all taxa.

## Conclusion

Our reanalysis of corrected data from four previously published studies on archosaur locomotion shows few substantive differences from our previously published results, although several reinterpretations of specific muscle functions have resulted. Nevertheless, these minor amendments do not undermine our earlier conclusions regarding the function and evolution of these locomotor systems and in some cases provide additional support for our previous interpretations. Our corrected data is available in the Supplemental Information for use in future studies on these interesting taxa.

## Supplemental Information

10.7717/peerj.1272/supp-1 Data S1 Corrected abduction–adduction and long axis rotation moment arm data

10.7717/peerj.1272/supp-2 Movie S1 Animation show hip flexion-extension motion, with the hip abduction–adduction fixed at 10°abduction and long-axis rotation fixed at zero

10.7717/peerj.1272/supp-3 Movie S2 Animation of abduction–adduction motion with the flexion-extension and long-axis rotation axes fixed at zero

10.7717/peerj.1272/supp-4 Movie S3 Animation of long-axis rotation motion with the flexion-extension and abduction–adduction axes fixed at zero

10.7717/peerj.1272/supp-5 Movie S4 Animation of abduction–adduction motion with the flexion-extension angle at 45° flexion and long-axis rotation axis fixed at zero

10.7717/peerj.1272/supp-6 Movie S5 Animation of long-axis rotation motion with the flexion-extension angle at 45° flexion and abduction–adduction axis fixed at zero
